# Growth Hormone, Insulin-Like Growth Factor-1, Insulin Resistance, and Leukocyte Telomere Length as Determinants of Arterial Aging in Subjects Free of Cardiovascular Diseases

**DOI:** 10.3389/fgene.2017.00198

**Published:** 2017-12-15

**Authors:** Irina D. Strazhesko, Olga N. Tkacheva, Dariga U. Akasheva, Ekaterina N. Dudinskaya, Ekaterina V. Plokhova, Valentina S. Pykhtina, Anna S. Kruglikova, Natalia V. Brailova, Natalia V. Sharashkina, Daria A. Kashtanova, Olesya Y. Isaykina, Mariya S. Pokrovskaya, Vladimir A. Vygodin, Irina N. Ozerova, Dmitry A. Skvortsov, Sergey A. Boytsov

**Affiliations:** ^1^Department of Clinical Cardiology and Molecular Genetics, Federal State Institution National Medical Research Center for Preventive Medicine of the Ministry of Healthcare of the Russian Federation, Moscow, Russia; ^2^Department of Age-associated Diseases, Medical Scientific and Educational Center, Lomonosov Moscow State University, Moscow, Russia; ^3^Russian Clinical Research Center for Gerontology, Pirogov Russian National Research Medical University, Moscow, Russia; ^4^Department of Fundamental and Applied Aspects of Obesity, Federal State Institution National Medical Research Center for Preventive Medicine of the Ministry of Healthcare of the Russian Federation, Moscow, Russia; ^5^Department of Cardiology, Federal Scientific and Clinical Center of the Federal Medico-Biological Agency, Moscow, Russia; ^6^Department of Aging and Age-associated Diseases Prevention, Federal State Institution National Medical Research Center for Preventive Medicine of the Ministry of Healthcare of the Russian Federation, Moscow, Russia; ^7^Department of Primary Prevention of Chronic Non-Communicable Diseases in the Healthcare System, Federal State Institution National Medical Research Center for Preventive Medicine of the Ministry of Healthcare of the Russian Federation, Moscow, Russia; ^8^Biobank, Federal State Institution National Medical Research Center for Preventive Medicine of the Ministry of Healthcare of the Russian Federation, Moscow, Russia; ^9^Department of Epidemiology of Chronic Non-Communicable Diseases Laboratory of Biostatistics, Federal State Institution National Medical Research Center for Preventive Medicine of the Ministry of Healthcare of the Russian Federation, Moscow, Russia; ^10^Department of Biochemical Markers of Chronic Non-Communicable Diseases Research, Federal State Institution National Medical Research Center for Preventive Medicine of the Ministry of Healthcare of the Russian Federation, Moscow, Russia; ^11^Department of Chemistry, Lomonosov Moscow State University, Moscow, Russia; ^12^National Medical Research Center for Cardiology of the Ministry of Healthcare of the Russian Federation, Moscow, Russia

**Keywords:** arterial aging, growth hormone, insulin-like growth factor-1, insulin resistance, leukocytes telomeres length

## Abstract

**Background:** Increased arterial stiffness (AS), intima-media thickness (IMT), and the presence of atherosclerotic plaques (PP) have been considered as important aspects of vascular aging. It is well documented that the cardiovascular system is an important target organ for growth hormone (GH) and insulin-like growth factor (IGF)-1 in humans, and GH /IGF-1 deficiency significantly increases the risk for cardiovascular diseases (CVD). The telomere length of peripheral blood leukocytes (LTL) is a biomarker of cellular senescence and that has been proposed as an independent predictor of (CVD). The aim of this study is to determine the role of GH/IGF-1, LTL and their interaction cardiovascular risk factors (CVRF) in the vascular aging.

**Methods:** The study group included 303 ambulatory participants free of known CVD (104 males and 199 females) with a mean age of 51.8 ± 13.3 years. All subjects had one or more CVRF [age, smoking, arterial hypertension, obesity, dyslipidemia, fasting hyperglycemia, insulin resistance—HOMA (homeostatic model assessment) >2.5, or high glycated hemoglobin]. The study sample was divided into the two groups according to age as “younger” (*m* ≤ 45 years, *f* ≤ 55 years) and “older” (*m* > 45 years, *f* > 55 years). IMT and PP were determined by ultrasonography, AS was determined by measuring the carotid-femoral pulse wave velocity (c-f PWV) using the SphygmoCor system (AtCor Medical). LTL was determined by PCR. Serum IGF-1 and GH concentrations we measured by immunochemiluminescence analysis.

**Results:** Multiple linear regression analysis with adjustment for CVRF indicated that HOMA, GH, IGF-1, and LTL had an independent relationship with all the arterial wall parameters investigated in the younger group. In the model with c-f PWV as a dependent variable, *p* < 0.001 for HOMA, *p* = 0.03 for GH, and *p* = 0.004 for LTL. In the model with IMT as a dependent variable, *p* = 0.0001 for HOMA, *p* = 0.044 for GH, and *p* = 0.004 for IGF-1. In the model with the number of plaques as a dependent variable, *p* = 0.0001 for HOMA, and *p* = 0.045 for IGF-1. In the older group, there were no independent significant associations between GH/IGF-1, LTL, HOMA, and arterial wall characteristics.

**Conclusions:** GH/IGF-1, IR, HOMA, and LTL were the important parameters of arterial aging in younger healthy participants.

## Introduction

Cardiovascular disease (CVD) remains a leading cause of death worldwide (Fuster et al., 2011). Steady aging of the population is leading to a significant increase in the number of heart attacks, strokes, and heart failures. More evidence has been obtained on the important prognostic implications of aging-related changes in the structural and functional parameters of the artery wall for the development of cardiovascular events. These changes create a metabolically and enzymatically active environment that promotes the onset and progression of disease.

Age-associated changes in the arteries include increased stiffness of large elastic arteries, for which the “gold standard” measurement method involves the carotid-femoral pulse wave velocity (c-f PWV) (Vlachopoulos et al., 2010). Other changes include an increase in the intima-media thickness (IMT) (Tziomalos et al., 2010) and the presence of atherosclerotic plaques (PP) (Green et al., 2011). Slowing these changes should reduce the risk of clinical CVD. The scientific basis for determining cardiovascular risk factors (CVRF) has increased significantly in recent years, but there is little information about the relationship between conventional risk factors and signs of arterial wall aging in individuals without clinical CVD. There is even less information on the association between signs of arterial wall aging and factors that determine the aging processes in general and in their interaction with CVRF. Factors that influence the aging process include insulin resistance (IR), growth hormone (GH) activity, insulin-like growth factor-1 (IGF-1), and leukocyte telomere length (LTL).

The levels of GH and IGF-1 decrease with age in both laboratory animals and humans (Carter et al., 2002). These hormones are important anabolics that stimulate cell growth, proliferation, and tissue repair. The age-associated decreases of GH and IGF-1 are believed to contribute to the development of many signs of aging, including cardiovascular dysfunction. Cardiomyocytes, smooth muscle cells, and endotheliocytes abundantly express IGF-1 receptors, which makes them highly sensitive to IGF-1 (Chisalita et al., 2009). People with GH deficiency and low circulating IGF-1 levels been documented to have an increased risk of developing CVD and cerebrovascular diseases (Laughlin et al., 2004).

There is a relationship between IR and arterial stiffness in both patients with diabetes and healthy young individuals (Giltay et al., 1999). The role of advanced glycation end-products and the effect of insulin could potentially be important in this context. IR also contributes to the progression of atherothrombosis through the development of dyslipidemia, hyperfibrinogenemia, and increased activity of tissue plasminogen activator inhibitor-1 (Choi et al., 2007).

Several studies have suggested that LTL may be an indicator of the biological age of vessels (Nilsson et al., 2013). Telomeres are the TTAGGG tandem repeats at the ends of chromosomes and progressively shorten with each replication of cultured human somatic cells. LTL reflects both an individual's telomere length at birth and the telomere attrition rates during the course of life, demonstrating the replicative history and cumulative oxidative burden (Aviv, 2004). Individuals with short telomeres in white blood cells are more likely to show accelerated vascular aging (Benetos et al., 2003), atherosclerosis (Samani et al., 2002), coronary heart disease (Fyhrquist et al., 2013) and type 2 diabetes mellitus (T2DM) (Murillo-Ortiz et al., 2012).

One of the least understood issues is the association between risk factors and arterial aging in different age groups. The predictive role of most CVRFs has been proven in studies involving mostly middle-aged people, and some of these factors are less significant in elderly people (Howard et al., 1997).

The aim of this study is to determine the role of GH/IGF-1, IR, and LTL in the vascular aging process and their interaction with CVRFs in subjects who are free of CVD.

## Materials and methods

The study included patients who had passed a preventive outpatient examination at the National Research Center for Preventive Medicine, Moscow, Russia, from 2012 to 2013. To determine eligibility, subjects completed a health screening which included their medical history, physical examination, and a blood sampling for laboratory analyses. We excluded 147 subjects with a previous history of drug medication for diabetes, hypertension, or hyperlipidemia; a history of stroke, coronary heart disease, peripheral arterial disease, arrhythmia, congestive heart failure, or valvular heart disease; hepatic or kidney failure; and cancer. Ultimately, 303 subjects were included in the study. All patients signed a legal informed consent form to participate in the study. The local ethics committee of the National Research Center for Preventive Medicine approved the study protocol.

Patients visited the clinic to undergo the study protocol examinations from 08:00 to 09:00 a.m. after a 12-h period of fasting. Blood pressure (BP) was measured using a brachial cuff (HEM-7200 M3, Omron Healthcare, Kyoto, Japan) on the right hand in a sitting position. The measurements were taken three times for 2-min intervals after a 10-min rest, and the average was used for analysis. Hypertension was diagnosed when BP was >140/90 mmHg.

During the cross-sectional study, anthropometric measurements were carried out with the calculation of the body mass index (BMI) in kg/m^2^. Obesity was diagnosed for BMI > 30.0 kg/m^2^. Abdominal obesity was indicated by a waist circumference (WC) >80 cm in women and >94 cm in men. The levels of total cholesterol (TC) and triglycerides (TG) were determined by a SAPPHIRE-400 biochemical analyzer (Niigata Mechatronics, Japan) with enzyme kits. The concentration of high-density lipoprotein cholesterol (HDL-C) was determined by the same analyzer in the supernatant after precipitation of serum ApoB-containing lipoproteins. The level of low-density lipoprotein cholesterol (LDL-C) was calculated by the Friedewald equation (when the TG level was no higher than 4.5 mmol/L). Hypercholesterolemia was diagnosed with TC > 5.0 mmol/L. HDL-C was considered to be decreased with a level of <1.2 mmol/L in women and <1.0 mmol/L in men. A level of TG > 1.7 mmol/l was considered to be elevated.

Serum fasting glucose (FG) was determined using routine laboratory methods with the biochemical analyzer. Fasting hyperglycemia was diagnosed when FG was ≥6.1 and <7.0 mmol/l. Serum insulin, GH and IGF-1 were quantified using the chemiluminescent microparticles on an immunoassay analyzer (Architect i 2000SR, Abbot, Canada). HOMA (homeostatic model assessment) was calculated as fasting insulin (mU/ml) × FG (mmol/l)/22.5. The level of glycated hemoglobin (HbA1c) was determined by the immunoturbodimetric method using the SAPPHIRE-400 analyzer.

T2DM was diagnosed when fasting glucose was ≥7.0 mmol/l and ≥11.1 mmol/l within 2 h after taking 75 g of glucose or HbA1c ≥ 6.5%. Participants were considered smokers if they had smoked more than 100 cigarettes during their entire life and if they smoked at the time of the study, whether every day or occasionally. Family history of CVD was considered if cases of CVD had been documented in first-degree relatives under the age of 65 years in women and 55 years in men. Age beyond 45 years for men and 55 years for women was considered a CVRF (National Cholesterol Education Program (NCEP) Expert Panel on Detection, 2002).

LTL was determined according to the method described by Cawthon (2002). Genomic deoxyribonucleic acid (DNA) was extracted directly from blood samples by standard procedures (OD_260nm/280nm_ 1.8–1.9). The assay involved comparing the abundance of telomere DNA to the single-copy genomic DNA number for each sample and by further comparison of the normalized values between DNA from different sources. The ratio of the telomere (T) and single-copy *36B4* gene matrices reflects the length of telomeres (the T/S ratio is approximately [$2_{\text{t}}^{\text{C}}$ (telomeres)/2^*C*^_t_(*36B4*)]^−1^ = 2^−Δ*C*^_t_ [T1]).

A 1.25× stock mix was also prepared [1× mixture: PCR buffer 1× (Fermentas 10× PCR Hotstartbuf + KCl), 2 mM MgCl_2_, 0.2 mM dNTP, 0.5 μM of each primer, 0.05 units/μl of Taq polymerase Maxima (Fermentas), and Sybr Green I 0.2×]. The primer sequences were the following: Tel1, GGTTTTTGAGGGTGAGGGTGAGGGTGAGGGTGAGGGT, Tel2, TCCCGACTATCCCTATCCCTATCCCTATCCCTATCCCTA, 36B4u, CAAGTGGGAAGGTGTAATCC, 36B4d, CCCATTCTATCATCAACGGGTACAA. Sixteen microliters of master mix were added to each sample well, along with 4 μl of the analyzed genomic DNA with a concentration of 10 ng/μl. Samples were mixed, centrifuged, and amplified in a CFX96 thermocycler.

For polymerase chain reaction (PCR) of the telomeres, we heated the samples at 95°C for 5 min and applied 35 cycles of 95°C for 20 s, and 54°C for 2 min, followed by melting. For the control PCR, we heated the samples at 95°C for 5 min and then applied 35 cycles of 95°C for 20 s, and 58°C for 1 min, followed by melting. The amplification of the corresponding telomeric and control mixtures occupied one cell unit. For each sample, we performed three repeated telomeric reactions and three control reactions. We calculated the difference between cycle thresholds of amplification of the telomere and single copy of the gene (ΔC_t_) and determined the relative telomere lengths based on the results.

The genomic DNA of the HEK cell line and a control leukocyte sample was used as a reference point. To take into account the differences in PCR mixtures from time to time, we set the leukocyte reference ΔC_t(leu)_ value at 8. The relative exponential length L was set as L = ΔC_t_-(ΔC_t(leu)_-8). We did not obtain the absolute value of the lengths of telomeres, so we used the standard deviation as a measure of the spread of values. In our experiment, the standard deviation in almost all cases was in the range of 0.1–0.4 derived from the relative lengths of 8.30–11.39 (logarithmic scale).

Arterial stiffness was assessed according to the c-f PWV values. It was measured using SphygmoCor 8.0 hardware (Atcor, Sydney) with the help of an applanation tonometer and electrocardiogram (ECG) gating to obtain pulse waves from both proximal sites (carotid artery) and distal sites (femoral artery). The c-f PWV was calculated from the transit time between the two sites relative to the R-wave within the electrocardiogram complex using the “foot-to-foot method” and the intersecting tangent algorithm (Rajzer et al., 2008). In each subject, two sequences of measurements were performed, and their mean was considered for analysis. The repeatability coefficient value was 0.935. Systolic blood pressure (SBP) and diastolic blood pressure (DBP) were measured with a manual sphygmomanometer. The mean of three consequent readings was recorded. PWV values >10 m/s were considered as elevated (Van Bortel et al., 2012).

Ultrasound examination of the carotid arteries was performed in B-mode by a linear high-resolution 17-5 MHz sensor (PHILIPS iU22, Netherlands). The studies were conducted by one operator. The atherosclerotic plaque was defined as thickening of focal vessel wall by more than 50% compared to the surrounding vessel wall areas or as a focal thickening of the intima-media complex by more than 1.3 mm protruding into the vessel lumen (Touboul et al., 2007). All measurements were performed in diastole, which corresponded to the R-wave on the electrocardiogram. PP was evaluated at six sites of the carotid arterial system: both common carotid arteries, both bifurcations, and both internal carotid arteries. The total number of all plaques was also determined. The average IMT was measured in automatic mode on both sides in the longitudinal section in the distal third of the common carotid at a distance of 1 cm proximal to the bifurcation. The posterior-wall IMT was measured as the distance between the vessel lumen and adventitia. The larger of the values on the right and left sides was taken into account. IMT values >0.9 mm were considered to indicate thickened walls (Perk et al., 2012).

### Statistical analysis

Statistical processing of the results was carried out using the statistical software package SAS 9.1 (SAS Institute, Cary, NC, USA). The mean value (M) and standard deviation (SD) were used to describe the quantitative indicators in the case of normal distributions, while the median and upper (UQ) and lower quartiles (LQ) were used in the case of abnormal distributions. To compare two groups with normal sample distribution the paired Student's *t*-test was used, for the abnormal distribution of variables the Mann-Whitney criterion was used. To compare the proportions Fisher's exact test was used. For the statistical description of the relationship between different parameters, Spearman's rank correlation analysis and intergroup comparison were conducted. Linear regression models were created to reveal the independence of the association between the risk factors and the parameters of the arterial wall. The vascular wall parameters were considered as dependent variables. The independent variables were indicators that demonstrated statistically significant associations with the vascular wall parameters in the correlation analysis and intergroup comparison. To assess the associations between the risk factors and the vascular wall parameters the logistic regression analysis was also used. Statistical significance level was set to *p* < 0.05.

## Results

We assumed that the nature of the relationship between the studied indicators and the artery wall parameters may differ between younger and older ages, which is why separate analyses were conducted for these groups. The younger group included men of 45 years of age and under and women of 55 years of age and under, while the older subjects formed the older group. The clinical characteristics of patients are presented in Table 1. In the older group, the SBP values were higher, the metabolic status indicators (WC, BMI, TC, LDL-C, TG, FG, HOMA) were worse, and levels of GH and IGFR-1 were lower, which are consistent with existing representations.

**Table 1:** Characteristics of the patients included in the study (*n* = 303).

**Characteristic**	**All patients (*n* = 303)**	**Younger group (*n* = 144)**	**Older group (*n* = 159)**	***p***
Age (years), *M* ±*SD*	51.5 ± 13.3	40.9 ± 8.7	61.1 ± 8.5	<0.001
Men (%)	34%	26.4	41.5	<0.001
Smoking (%)	19.1	20.8	17.6	0.478
Arterial hypertension (%)	25.4	17.4	32.0	0.003
Hypercholesteremia (%)	66.3	61.1	71.1	0.068
Fasting hyperglycaemia (%)	22.4	10.4	33.3	<0.001
T2DM (%)	16.5	5.6	26.4	<0.001
Obesity (%)	24.4	23.6	25.2	0.754
Family history of CVD (%)	21.0	24.3	18.0	0.182
SBP (mmHg), *M* ±*SD*	125.4 ± 16.4	120.6 ± 15.3	129.7 ± 16.2	<0.001
DBP (mmHg), *M* ±*SD*	78.2 ± 10.2	77.5 ± 10.4	78.9 ± 10.1	0.250
WC (cm), *M* ±*SD*	89.6 ± 15.3	85.8 ± 15.3	93 ± 14.5	<0.001
BMI (kg/m2), *M* ±*SD*	27.4 ± 5.1	26.7 ± 5.6	27.9 ± 4.6	0.041
TC (mmol/l), *M* ±*SD*	5.7 ± 1.2	5.4 ± 1.0	5.9 ± 1.2	<0.001
LDL-C (mmol/l), *M* ±*SD*	3.9 ± 1.1	3.7 ± 0.9	4.0 ± 1.4	0.008
HDL-C (mmol/l), *M* ±*SD*	1.2 ± 1.3	1.2 ± 0.3	1.2 ± 0.3	0.208
TG (mmol/l), Med (LQ-UQ)	1.04 (0.76–1.51)	0.92 (0.63–1.37)	1.2 (0.86–1.67)	<0.001
Fasting glucose (mmol/l)	5.8 ± 1.4	5.3 ± 1.0	6.1 ± 1.6	<0.001
HOMA, Med (LQ-UQ)	1.83 (1.31–2.93)	1.7 (1.19–2.52)	2.03 (1.41–3.22)	0.01
HbA1c (%), *M* ±*SD*	5.48 ± 0.96	5.17 ± 0.71	5.77 ± 1.06	<0.001
Insulin (mIU/L), *M* ±*SD*	9.07 ± 4.71	8.32 ± 4.66	10.06 ± 5.69	0.134
CRP (mg/l), Med (LQ-UQ)	2.3 (1.6–3.9)	2.1 (1.5–3.1)	2.7 (1.7–4.2)	0.257
GH, ng/ml, Med (LQ-UQ)	0.49 (0.12–2.0)	0.67 (0.11–2.84)	0.39 (0.12–1.06)	0.006
IGF-1, ng/ml, *M* ±*SD*	150.8 ± 58.6	161.9 ± 63.2	141 ± 52.6	0.004
LTL, *M* ±*SD*	9.77 ± 0.50	9.93 ± 0.48	9.63 ± 0.46	<0.001

*BMI, body mass index; CRP, high sensitive C-reactive protein; CVD, cardiovascular diseases; DBP, diastolic blood pressure; FG, fasting glucose; GH, growth hormone; HbA1c, glycated hemoglobin; HDL-C, cholesterol of high-density lipoproteins; HOMA, insulin resistance index; IGF-1, insulin-like growth factor 1; LDL-C, cholesterol of low-density lipoproteins; LTL, leukocytes telomere length; SBP, systolic blood pressure; TC, total cholesterol; TG, triglycerides; T2DM, diabetes mellitus type 2; WC, waist circumference; p, p-value between younger and older groups*.

A comparison of the arterial wall characteristics in both groups is presented in Table 2. In the older group, the arterial stiffness and IMT were higher, there were more atherosclerotic plaques, and values of the studied parameters associated with an increased risk of CVD were more common. The results of Spearman's correlation analysis of the association of arterial wall parameters and conventional CVRF, GH, IGF-1, LTL, and HOMA are presented in Table 3. In the general group, the arterial wall characteristics are associated with almost all studied parameters.

**Table 2:** The characteristics of arterial wall in both age groups (*n* = 303).

	**All patients (*n* = 303)**	**Younger group (*n* = 144)**	**Older group (*n* = 159)**	***p***
c-f PWV (m/s), *M* ±*SD*	8.8 ± 2.1	7.9 ± 1.7	9.6 ± 2.1	<0.001
c-f PWV > 10 m/s (*n*, %)	88 (31.2)	20 (14.9)	68 (46)	<0.001
IMT (mm), *M* ±*SD*	0.75 ± 0.19	0.64 ± 0.15	0.86 ± 0.17	<0.001
IMT > 0.9 mm (*n*,%)	67 (23.5)	11 (8.1)	56 (37.3)	<0.001
AP (*n*), *M* ±*m*	1.09 ± 0.09	0.35 ± 0.07	1.77 ± 0.13	<0.001
PP (*n*, %)	138 (48.3)	30 (22.1)	108 (72)	<0.001

*AP, atheroslerotic plaques; IMT, intima-media thickness; c-f PWV, carotid-femoral pulse wave velocity; PP, plaques presence; p, p-value when comparing the younger and older groups*.

**Table 3:** Association between the arterial wall parameters and studied indicators in the general group.

	**c-f PWV**	**IMT**	**AP**
Age	0.45 (*p* < 0.001)	0.68 (*p* < 0.001)	0.58 (*p* < 0.001)
SBP	0.35 (*p* < 0.001)	0.39 (*p* < 0.001)	0.36 (*p* < 0.001)
DBP	0.22 (*p* < 0.001)	0.18 (*p* = 0.003)	0.16 (*p* = 0.008)
WC	0.31 (*p* < 0.001)	0.34 (*p* < 0.001)	0.20 (*p* < 0.001)
BMI	0.28 (*p* < 0.001)	0.29 (*p* < 0.001)	0.17 (*p* = 0.003)
TC	0.22 (*p* < 0.001)	0.25 (*p* < 0.001)	0.21 (*p* < 0.001)
LDL-C	0.17 (*p* = 0.004)	0.20 (*p* < 0.001)	0.18 (*p* = 0.003)
HDL-C	−0.13 (*p* = 0.028)	−0.16 (*p* = 0.007)	−0.12 (*p* = 0.04)
TG	0.38 (*p* < 0.001)	0.44 (*p* < 0.001)	0.30 (*p* < 0.001)
FG	0.37 (*p* < 0.001)	0.35 (*p* < 0.001)	0.31 (*p* = 0.015)
HOMA	0.33 (*p* < 0.001)	0.29 (*p* < 0.001)	0.18 (*p* = 0.003)
GH	−0.17 (*p* = 0.007)	−0.13 (*p* = 0.034)	0.006 (*p* = 0.929)
IGF-1	−0.05 (*p* = 0.462)	−0.023 (*p* < 0.001)	−0.22 (*p* < 0.001)
LTL	−0.33 (*p* < 0.001)	−0.30 (*p* < 0.001)	−0.29 (*p* < 0.001)

*The results of the Spearman's correlation analysis (n = 303). BMI, body mass index; DBP, diastolic blood pressure; FG, fasting glucose; GH, growth hormone; HDL-C, cholesterol of high-density lipoproteins; HOMA, insulin resistance index; IGF-1, insulin-like growth factor 1; LDL-C, cholesterol of low-density lipoproteins; LTL, leukocytes telomere length; SBP, systolic blood pressure; TC, total cholesterol; TG, triglycerides; WC, waist circumference; p, p-value between younger and older groups*.

Taking into account the available data on the decrease of the role of conventional CVRF with age (Howard et al., 1997), we separately analyzed the relationship between the studied parameters and the vascular wall characteristics in the two age groups (see Table 4). Notably, almost all parameters demonstrated an association with the arterial wall characteristics in the younger group. However, some of them lost the connection with arterial wall characteristics in the older group. This also applies to GH, IGF-1, and HOMA. The strength of the association, even if it is preserved, is weaker in the older group.

**Table 4:** Associations between the arterial wall characteristics and studied parameters in different age groups.

	**c-f PWV**		**IMT**		**AP**	
	**Younger group**	**Older group**	**Younger group**	**Older group**	**Younger group**	**Older group**
Age	0.48 (*p* < 0.001)	0.05 (*p* = 0.477)	0.52 (*p* < 0.001)	0.43 (*p* < 0.001)	0.43 (*p* < 0.001)	0.27 (*p* < 0.001)
SBP	0.25 (*p* = 0.003)	0.21 (*p* = 0.011)	0.38 (*p* < 0.001)	0.23 (*p* = 0.004)	0.25 (*p* = 0.003)	0.29 (*p* < 0.001)
DBP	0.31 (*p* < 0.001)	0.07 (*p* = 0.371)	0.35 (*p* < 0.001)	0.04 (*p* = 0.645)	0.20 (*p* = 0.020)	0.15 (*p* = 0.074)
WC	0.29 (*p* < 0.001)	0.12 (*p* = 0.131)	0.47 (*p* < 0.001)	0.02 (*p* = 0.767)	0.21 (*p* = 0.016)	−0.02 (*p* = 0.8)
BMI	0.36 (*p* < 0.001)	0.12 (*p* = 0.157)	0.46 (*p* < 0.001)	0.05 (*p* = 0.562)	0.20 (*p* = 0.019)	0.02 (*p* = 0.8)
TC	0.30 (*p* < 0.001)	0.06 (*p* = 0.456)	0.36 (*p* < 0.001)	0.05 (*p* = 0.561)	0.20 (*p* = 0.018)	0.10 (*p* = 0.240)
LDL-C	0.25 (*p* = 0.004)	0.02 (*p* = 0.807)	0.33 (*p* < 0.001)	−0.02 (*p* = 0.849)	0.18 (*p* = 0.033)	0.06 (*p* = 0.437)
HDL-C	−0.12 (*p* = 0.157)	−0.05 (*p* = 0.567)	−0.27 (*p* = 0.001)	−0.03 (*p* = 0.747)	−0.18 (*p* = 0.033)	−0.04 (*p* = 0.665)
TG	0.43 (*p* < 0.001)	0.22 (*p* = 0.006)	0.60 (*p* < 0.001)	0.22 (*p* = 0.009)	0.31 (*p* < 0.001)	0.12 (*p* = 0.158)
FG	0.33 (*p* < 0.001)	0.23 (*p* = 0.004)	0.21 (*p* = 0.015)	0.20 (*p* = 0.015)	0.18 (*p* = 0.038)	0.18 (*p* = 0.025)
HOMA	0.35 (*p* < 0.001)	0.24 (*p* = 0.005)	0.39 (*p* < 0.001)	0.14 (*p* = 0.102)	0.21 (*p* = 0.017)	0.11 (*p* = 0.19)
GH	−0.23 (*p* = 0.014)	−0.008 (*p* = 0.919)	−0.27 (*p* = 0.003)	0.06 (*p* = 0.476)	−0.001 (*p* = 0.989)	0.06 (*p* = 0.505)
IGF-1	−0.12 (*p* = 0.200)	−0.15 (*p* = 0.081)	−0.34 (*p* < 0.001)	−0.05 (*p* = 0.556)	−0.27 (*p* = 0.003)	−0.10 (*p* = 0.209)
LTL	−0.36 (*p* = 0.0001)	−0.15 (*p* = 0.112)	−0.08 (*p* = 0.543)	−0.06 (*p* = 0.434)	−0.13 (*p* = 0.137)	−0.03 (*p* = 0.637)

*The result of the Spearman's correlation analysis*.

*BMI, body mass index; DBP, diastolic blood pressure; FG, fasting glucose; GH, growth hormone; HDL-C, cholesterol of high-density lipoproteins; HOMA, insulin resistance index; IGF-1, insulin-like growth factor 1; LDL-C, cholesterol of low-density lipoproteins; LTL, leukocytes telomere length; SBP, systolic blood pressure; TC, total cholesterol; TG, triglycerides; WC, waist circumference*.

To identify the independent relationships of some of the clinical and biological factors with c-f PWV, IMT, atherosclerotic plaque number (APN), linear regression models were compiled using the arterial wall indicators as a dependent variable. Parameters that demonstrated a statistically significant relationship with the vascular wall in the correlation analysis were used as independent variables. Thus, to create a forecast model for each dependent variable, a set of explanatory variables was formed. Only statistically significant variables were presented in the final regression equation. Considering the previously identified differences between the younger and older groups in listed factors associated with a particular arterial wall parameter, the regression models were performed for not only the general group, but separately for older and younger groups.

In the general group, only c-f PWV as a dependent variable was associated with GH, IGF-1, and LTL (see Table 5). A model that evaluated age, HbA_1c_, LTL, HOMA, GH, and IGF-1 explained 36% of the c-f PWV variability. IMT and the APN did not demonstrate this relationship. In the younger group, HOMA, GH, and IGF-1 demonstrated an independent relationship with all the studied arterial wall parameters, but LTL only had a relationship with c-f PWV. The highest coefficient of *R*^2^ = 0.45 was in the IMT variability model. The results of multiple linear regression analysis (with adjustment for CVRF) in the younger group are presented in Table 6. In contrast, there were no independent significant associations of GH/IGF-1 and arterial wall characteristics obtained through multiple linear regression analysis in the older group.

**Table 5:** Stepwise multiple linear regression analysis of c-f PWV as dependent variable in general group (with adjustment for CVRF).

**Predictor**	**β ±*SE***	**Type II SS**	***p***	***R*^2^**
Intercept	12.569 ± 3.852	48.853	0.001	
Age	0.076 ± 0.013	146.890	0.0001	
HbA1c	0.408 ± 0.167	27.438	0.015	
HOMA	0.182 ± 0.075	26.985	0.016	
GH	−0.153 ± 0.065	25.986	0.018	
IGF-1	−0.005 ± 0.003	29.118	0.013	
LTL	0.896 ± 0.356	17.424	0.053	0.3595

*GH, growth hormone; HbA1c, glycated hemoglobin; HOMA, insulin resistance index; IGF-1, insulin-like growth factor 1; LTL, leukocytes telomere length; S.E., Standard error; Type II SS, type II sum of squares*.

**Table 6:** Stepwise multiple linear regression analysis (with adjustment for CVRF) of c-f PWV, IMT, Number of Plaques as dependent variables in the younger group.

**Predictor**	**β ±*SE***	**Type II SS**	***P***	**Model *R*^2^**
**c-f PWV – DEPENDENT VARIABLE**				
Intercept	22.871 ± 4.678	70.130	0.0001	
HOMA	0.371 ± 0.108	34.541	<0.001	
GH	−0.138 ± 0.062	14.185	0.03	
LTL	−1.370 ± 0.468	25.130	0.004	0.2868
**IMT – DEPENDENT VARIABLE**				
Intercept	0.582 ± 0.052	1.430	0.0001	
GH	−0.009 ± 0.005	0.044	0.056	
IGF-1	−0.001 ± 0.0001	0.102	0.004	
HOMA	0.041 ± 0.007	0.399	0.0001	0.4528
**NUMBER OF PLAQUES – DEPENDENT VARIABLE**				
Intercept	0.254 ± 0.198	0.641	0.203	
IGF-1	−0.002 ± 0.001	1.615	0.045	
HOMA	0.192 ± 0.034	12.130	0.0001	0.2920

*GH, growth hormone; IMT, intima-media thickness; HbA1c, glycated hemoglobin; HOMA, insulin resistance index; IGF-1, insulin-like growth factor 1; LTL, leukocytes telomere length; c-f PWV, carotid-femoral pulse wave velocity; SE, Standard error; Type II SS, type II sum of squares*.

A logistic regression method was used to evaluate the associations between the studied parameters and vascular wall characteristics. The selection of predictors was carried out based on the linear regression models. The method of stepwise exclusion of predictors was used. Only statistically significant predictors (*p* < 0.05) were retained in the final model. The relative contribution of individual predictors was determined by the value of the Wald chi-squared statistics. Binary indicators based on median hormone levels were used as characteristics of the hormonal status: the median GH was 0.50 ng/ml and the median IGF-1 was 140 ng/ml. The median of individual LTL values was 9.75. Telomeres were considered to be short if their length was less than this value and long if TL ≥ 9.75. Long telomeres were observed in 156 people, while 141 people had short telomeres. Telomeres were considered to be the shortest if LTL was <9.25, which corresponded to the first quartile of distribution (Q1) (*n* = 35). Telomeres were considered the longest with LTL > 10.25 (IV quartile – QIV) (*n* = 42).

Logistic regression analysis in the general group showed that the probability of having stiff arteries (c-f PWV > 10m/s) increased with HOMA > 2.5 and the presence of short telomeres (Table 7). In the younger group the results of logistic regression analysis were statistically significant for only the PP. The probability of having atherosclerotic plaques was increased in Q1 TL. IGF-1 greater than the median was associated with a five-fold lower probability of atherosclerotic plaques being present (Table 8).

**Table 7:** Results of logistic regression analysis in general group where c-f PWV > 10 m/s is dependent variable.

**Predictor**	**β ±*SE***	**χ^2^ Wald statistic**	***p***	**OR**	**95% CI**
**Model 1. Predictors: Old age, male sex, ↑SBP, ↑HOMA, Fasting hyperglycemia, ↑TG, LTL<9.75**					
Old age	1.521 ± 0.330	21.330	0.0001	4.58	2.40–8.73
↑HOMA	0.658 ± 0.319	4.261	0.039	1.93	1.03–3.61
LTL < 9.75	0.893 ± 0.313	8.141	0.004	2.44	1.32–4.51

*HOMA, insulin resistance index; LTL, leukocytes telomere length*.

**Table 8:** Results of logistic regression analysis in younger group, where the presence of AP is a dependent variable.

**Predictor**	**β ±*SE***	**χ^2^ Wald statistic**	***p***	**OR**	**95% CI**
**Model 3. Predictors: male sex, smoking, ↑SBP, ↑HbA1c, ↑HOMA, ↑BMI, ↑CRP, IGF-1>Med, Q1TL**					
↑CRP	2.737 ± 0.897	9.304	0.002	15.44	2.66–89.63
Q1LTL	2.832 ± 1.066	7.059	0.008	16.98	2.10–137.01
IGF-1>Med	−1.75 ± 0.731	5.725	0.017	0.174	0.04–0.73

*BMI, body mass index; CRP, high sensitive C-reactive protein; HbA1c, glycated hemoglobin; HOMA, insulin resistance index; Med, median; SBP, systolic blood pressure; Q1LTL, LTL was <9.25, which corresponded to the first quartile of distribution*.

The results indicate that the level of hormones (GH, IGF-1) and HOMA index were significantly related to the vascular wall parameters in the general and younger groups but not the older group. These data once again confirm the idea of a decrease in the role of certain factors in the older group in comparison with the younger one. Differences between older and younger people can be explained by the fact that people whose risk factors led to subclinical changes at a younger age after reaching old age had clinical manifestations of CVD and could not become participants in our study.

GH was associated with c-f PWV and IMT, while IGF-1 was associated with APN and IMT. The association of these hormones with IMT was higher than with other parameters of the vascular wall. It is important that the negative relationship testifies to the protective role of GH and IGF-1 for pathological changes in the arteries. The HOMA index was associated with all arterial wall parameters. LTL is associated with both increased arterial stiffness and subclinical atherosclerosis. It can be traced both in the younger and general groups. The obtained results determine potential therapeutic targets to prevent arterial changes.

## Discussion

We consider that the first important result of this work is the identification of the significant association of GH/IGF- 1 (independently of CVRF) with the main arterial wall parameters in healthy people without hypopituitarism (HP). The role of IGF-1 in cardiovascular pathology was firstly observed in studies of the causes of death in patients with pituitary diseases. Epidemiological studies have shown that in patients with a long period of IGF-1 deficiency and somatotropic insufficiency, cardiovascular mortality was two times higher than in the general population (Higashi et al., 2012). GH/IGF-1 deficiency leads to physiological age-related changes in the cardiovascular system, such as a decrease in the cardiomyocyte number, fibrosis, collagen accumulation, and decreases in the synthesis of proteins, including contractile actin and myosin (LeRoith et al., 1995).

Rosén and Bengtsson (1990) were the first to demonstrate a reduction in the quality of life in patients with HP. They analyzed data from 333 patients with HP who visited endocrinology clinics between 1956 and 1987. During the follow-up period, 104 patients died, which corresponded to a significantly higher mortality rate compared to the general population. The excess mortality was related to deaths from CVD, and the most common causes were myocardial infarction (MI), coronary artery disease (CAD), congestive heart failure, and cerebrovascular disease. Subsequently, the relationship between the level of GH/IGF-1 and the prognosis of CVD was revealed in non-HP patients.

In a cross-sectional study, Spallarossa et al. showed that a low level of IGF-1 was associated with angiographically confirmed CAD (Spallarossa et al., 1996). A prospective case-control study observed 600 participants for 15 years and showed that the level of IGF-1 below the median increased the risk of CAD (Juul et al., 2002). The level of IGF-1 predicted death from CAD in 1,185 people of both sexes in a study conducted by Laughlin et al. (2004) A low level of IGF-1 was associated with a worse prognosis in the early period of MI (Conti et al., 2001).

The explanation of these relationships may lie in the connection of the hormone levels with the arterial wall, even at subclinical changes. Thus, flow-dependent vasodilatation of peripheral arteries was impaired in patients with GH deficiency (Smith et al., 2002). Galderisi et al. revealed a positive relationship between IGF-1 and coronary reserve (Galderisi et al., 2002). A cross-sectional study on 400 elderly men conducted by van Den Beld et al. revealed an inverse correlation between IGF-1 and IMT (van den Beld et al., 2003). These results become clear when considering that the cardiovascular system is the target of GH and IGF-1. Smooth muscle cells (SMCs) and endotheliocytes abundantly express IGF-1 receptors (Chisalita and Arnqvist, 2004). IGF-1 is a powerful mitogenic, anti-apoptotic, and promigrant factor for both endotheliocytes and SMC (Arnqvist, 2008). That is, IGF-1 can be pro-atherogenic by its ability to stimulate the migration and proliferation of SMC and macrophage migration (Renier et al., 1996) to promote the expression of adhesion molecules (Li et al., 2009). On the other hand, a decrease in the level of IGF-1 can cause plaque destabilization (Libby and Sasiela, 2006).

In our study, IGF-1 apparently played a protective role in plaque formation, arterial wall thickening, and increasing stiffness. These effects can be associated with pronounced anti-inflammatory and antioxidant properties of IGF-1 and its ability to enhance reparative mechanisms (Sukhanov et al., 2007), primarily in the endothelium. Most clinical studies support the concept that normal levels of GH and IGF-1 are necessary to maintain endothelial health. IGF-1 is involved in the synthesis of nitrogen monoxide (NO) in endothelial cells, causing additional vasodilation of the arteries. This leads to a decrease in the concentration of free fatty acids (FFA) and an increase in sensitivity to native insulin. Our studies are consistent with others indicating that patients with GH deficiency and low circulating IGF-1 had an increased risk of CVD (Vasan et al., 2003; Conti et al., 2004; Laughlin et al., 2004).

It is impossible not to recall contradictory results on the role of GH/IGF-1 in life expectancy. In some, not all, studies on rodents, it was shown that a decrease in the activity of GH/IGF-1 leads to an increase in life expectancy (Brown-Borg et al., 1996; Coschigano et al., 2000; Flurkey et al., 2001). The most important question remains the following: “Does the deficiency of these hormones in humans increase the life expectancy like animals?” It should be noted that although there are isolated cases when patients with dwarfism lived longer than their peers, the overall life expectancy of people with dwarfism did not differ from healthy ones, which was shown in patients with Laron's syndrome (a hereditary disease with an autosomal recessive type of inheritance-variety dwarfism caused by a congenital defect in the GHR receptor gene), leading to insensitivity of peripheral tissues to the action of GH (Laron, 2005). A meta-analysis of 12 studies, including 14,906 people, showed that there is a U-shaped curve of the relationship between the level of IGF-1 and overall mortality. Low levels of IGF-1 are associated with higher mortality due to the increase in cardiovascular mortality, high IGF-1 values are associated with higher cancer mortality (Burgers et al., 2011). Thus, the data from clinical studies do not support the concept of the role of GH/ IGF-1 in increasing the life expectancy that was observed in invertebrates and rodents. It can be assumed that the signaling pathway GH/ IGF-1 is not an evolutionarily conservative mechanism for regulating life expectancy. At the same time, this signaling pathway plays an important role in the development and prevention of age-associated diseases.

The second important result of this work is the detection of a negative relationship between the HOMA index and arterial wall parameters. This can be explained by the fact that despite the PI3K-Akt signaling pathway blockade in IR, the pathway of mitogen-activating protein kinases (MAPK), which does not depend on the receptor sensitivity to insulin, continues to function. This stimulates the SMC proliferation and migration and causes a prothrombotic state. Compensatory hyperinsulinemia accompanying IR shifts the balance of signaling pathways toward mitogenic action, which promotes an accelerated atherogenesis. The stimulation of insulin by the local renin-angiotensin system of blood vessels causes an increase in NADPH-oxidase activity, a decrease in the bioavailability of NO, and an increase in the production of reactive oxygen species (ROS) (Wang et al., 2007).

In IR angiotensin II, oxidative stress, endothelial dysfunction, pro-inflammatory cytokines, and adhesion molecules activate matrix metalloproteinases (MMPs), which cause fragmentation of elastin molecules and increase collagen stiffness (Jacob, 2003). As the number of collagen molecules increases, collagen binds to glucose molecules with the formation of cross-links represented by advanced glycation end products (AGEs), which significantly increase collagen rigidity and disrupt normal processes of its transformation. Activated MMPs promote basal membrane degradation as well as enhance the migration of SMCs and intimal proliferation (Wang and Lakatta, 2002).

IR is characterized by the development of dyslipidemia, which is an increase in TG and LDL, as well as a decrease in the level of HDL. LDL is mainly represented by the subfraction of highly atherogenic dense particles. Their ability to bind to LDL receptors is reduced, so they circulate for a long time in the bloodstream, become oxidized, and are actively captured by macrophages. Macrophages secreting growth factors and cytokines cause a thickening of the vessel wall and contribute to the plaque development and destabilization (Ford et al., 2002). It was shown that hyperinsulinemia caused hyperfibrinogenemia and an increase in the activity of plasminogen activator inhibitor-1, which led to fibrinolysis failure. Violations in the fibrinolysis system contribute to the progression of atherothrombosis (Choi et al., 2007).

The third important result of this work is the detection of the independent inverse relationship of LTL with both increased arterial stiffness (arteriosclerosis) and atherosclerosis. It is well-known, that the process of vascular aging is characterized, even in apparently healthy subjects, by number of deleterious changes within the vascular wall that are involved both in atherosclerosis and arterial stiffening (Palombo and Kozakova, 2016). Aging is associated with endothelial dysfunction, decreased bioavailability of NO, increased bioavailability of ROS as well as with low-grade inflammation. Age also induces degradation and fragmentation of elastic fibers and a non-enzymatic cross-linkage between collagen fibers. The functional capacity of stem and progenitor cells play key role in these processes. These cells participate in damage repair and tissue differentiation processes, and they play an important role in maintaining tissue homeostasis, including the vessel wall (Sharpless and DePinho, 2007). In clinical practice, the length of telomeres is determined in leukocytes and it reflects the length of telomeres in stem cells and progenitor cells. There is increasing evidence that low telomerase activity and telomere shortening are key components of the reduction in stem cell reserves, age-associated tissue degeneration, and increased vascular stiffness (Sharpless and DePinho, 2007). We have shown that the presence of short telomeres with a length corresponding to the first quartile of the distribution was associated with 17 times higher probability of the presence of AP in the younger group. These data are consistent with the results of the latest meta-analyses, which proved that short telomeres were associated with atherosclerotic CVD (D'Mello et al., 2015). Moreover, it is now widely acknowledged that LTL is not a passive marker but an active determinant of atherosclerosis development, since it determines the replicative and reparative abilities of tissue (Calado and Young, 2009) in response to the influence of risk factors.

Another interesting result is the differences revealed in the relationship of risk factors and arterial wall condition in different age groups. Our study has shown that the value of traditional risk factors for the vascular wall changes was reduced in the older group. Similar results indicating a decrease in the association of conventional risk factors and subclinical changes in arteries in older age were obtained in the Cardiovascular Health Study and the Atherosclerosis Risk in Communities Study (Howard et al., 1997).

The level of lipids and BMI in the elderly was significantly less associated with CVD risk than in the young (Psaty et al., 1999). Obesity and hyperlipidemia in the older group were recognized as unimportant risk factors. In the Italian longitudinal study of aging the metabolic syndrome was not associated with a risk of MI and stroke in the elderly (Motta et al., 2009) According to other data, an increase in the BMI to 27.0 in the elderly did not result in an increase in the number of cardiovascular events and mortality from all causes (Heiat et al., 2001). It was suggested that elderly people with risk factors but not having clinical CVD are resistant to the influence of CVRF.

## Conclusion

GH/IGF-1 along with IR and LTL play important roles in the development of arterial aging. The negative relationship between GH/IGF-1 and arterial wall characteristics attests to the protective role of these hormones in arterial wall changes. The arterial walls should be evaluated at a young age, even in the absence of clinical manifestations of CVD and primarily in people with CVRF. To predict changes in the vascular wall, it is advisable to study not only conventional risk factors, but also indicators such as GH/IGF-1, LTL, and HOMA.

## Author contributions

IS, OT, NB, NS, OI, DK, VV, IO, MP, DS, SB: Creation of the conception and design of the work, revision the work, final approval of the version to be published, agreement for all aspects of the work. DA, ED, EP, VP, AK, MP, DS: Acquisition, analysis, interpretation of data for the work, revision the work, final approval of the version to be published, agreement for all aspects of the work.

### Conflict of interest statement

The authors declare that the research was conducted in the absence of any commercial or financial relationships that could be construed as a potential conflict of interest.
